# A Case of Acute Pancreatitis Developing After Upper Gastrointestinal Endoscopy: A Rare Post-Procedural Complication

**DOI:** 10.7759/cureus.98812

**Published:** 2025-12-09

**Authors:** Hasan H Çoban, Güngör Sitar, Elif N Yilmaz, Semih Eker, Ayşenur Ünlütürk

**Affiliations:** 1 Internal Medicine, University of Health Sciences, Sancaktepe Şehit Prof. Dr. İlhan Varank Training and Research Hospital, Istanbul, TUR

**Keywords:** acute pancreatitis (ap), gastritis, gi endoscopy, polypectomy, rare complication

## Abstract

Acute pancreatitis is a rare but clinically significant complication of upper gastrointestinal endoscopy. Its diagnosis can be easily overlooked if abdominal symptoms are associated with the primary indication for the procedure. Although endoscopy is generally considered a reliable diagnostic tool, postprocedural pancreatitis has rarely been described in the literature, and its underlying mechanisms are not yet fully understood. Possible explanations include mechanical irritation from gastric or duodenal manipulations, increased intraluminal pressure due to air inflation, or induction of procedure-related pancreatic duct obstruction in susceptible patients. We report the case of a 57-year-old woman who underwent upper gastrointestinal endoscopy because of persistent vomiting and increasingly severe abdominal pain for a week. On presentation, she complained of a streaky, posteriorly directed upper abdominal pain and recurrent nausea and vomiting. Laboratory tests revealed elevated lipase (264.8 U/L; reference range: 13-60) and amylase (201 U/L; reference range: 28-100). Physical examination revealed marked tenderness in the upper abdomen without guarding or rebound tenderness. Imaging studies confirmed the diagnosis of acute pancreatitis. The patient was admitted to the internal medicine department and treated conservatively with analgesics, fluid therapy, and bowel rest. Her symptoms gradually improved, and she was discharged home in stable condition. This case highlights the importance of upper gastrointestinal endoscopy as a possible (albeit rare) trigger for acute pancreatitis, especially when new or worsening abdominal pain occurs shortly after the procedure. Given the limited number of published cases and the uncertainty of causal relationships, documentation of such cases is important to increase clinical awareness, guide diagnostic suspicion, and contribute to a better understanding of this rare complication.

## Introduction

Acute pancreatitis originates from a variety of aetiologies, including gallstones, alcohol abuse, hypertriglyceridemia, medications, autoimmune conditions, and invasive procedures. Diagnosis is based on at least two of the following Atlanta criteria: characteristic abdominal pain, serum lipase or amylase levels ≥3 times the upper limit of normal, and radiographic evidence of pancreatitis [1]. Initial treatment includes bowel rest, intravenous hydration, and gradual reintroduction of oral intake. Further imaging is often indicated [2].

Although upper gastrointestinal endoscopy is generally considered a safe diagnostic procedure, acute pancreatitis occurring after endoscopy is exceedingly rare, and its mechanism is not fully understood. Proposed contributors include prolonged procedure time, repeated repositioning, and excessive air insufflation, all of which may result in mechanical or pressure-related pancreatic irritation.

This report documents a rare case of acute pancreatitis following endoscopy in a patient without the usual risk factors. It aims to raise clinical awareness of this unusual complication and to highlight the importance of endoscopy as a potential trigger when new abdominal symptoms arise shortly after the procedure.

## Case presentation

A 57-year-old woman (155 cm, 65 kg) with a history of unipolar depression since 2014 and hypertension since 2023, presented to the internal medicine outpatient clinic in November 2024. The patient had no history of diabetes mellitus, alcohol use, pancreatic cancer, or pancreatitis, and no relevant family history. There was also no occupational exposure to chemicals or toxins. Her antihypertensive therapy included candesartan/hydrochlorothiazide 32 mg/12.5 mg. With the addition of amlodipine 10 mg due to inadequate blood pressure control, normotension was achieved at the follow-up. In addition, the patient had been experiencing intermittent vomiting for three years, and the attacks usually recurred several times a week and usually occurred during stressful periods, lasted approximately 10-15 minutes, and were accompanied by the sudden onset of vomiting. This was accompanied by mild nausea during the day, which negatively affected daily mood. The patient's unipolar depression was treated with vortioxetine. Requested due to the gastrointestinal symptoms, an upper gastrointestinal endoscopy procedure lasting approximately 30 minutes was performed approximately one week prior to admission, and two biopsy samples were taken during the procedure. Difficulty was experienced, especially during polyp removal, and the patient's stretcher had to be elevated to approximately 45 degrees for optimal positioning. CO_2_ insufflation was maintained throughout the procedure.

During endoscopy, biopsies were taken from hyperemic and edematous antral mucosa, and a small polyp in the cardia was removed with forceps. The procedure was concluded with a diagnosis of antral gastritis and a cardia polyp. Histopathological evaluation of the antral biopsy revealed chronic inactive gastritis with positive inflammatory activity, and the cardia polyp specimen showed focal edema, mild bleeding, and polypoid gastric corpus mucosa with mild chronic inflammation beneath the surface epithelium, consistent with chronic inactive gastritis. Seven days after endoscopy, the patient was admitted to the internal medicine department because of persistent vomiting, worsening abdominal pain compared to preprocedural levels, and elevated pancreatic enzymes. Lipase was measured at 264.8 U/L (U/L: enzyme activity units per liter) and amylase at 201 U/L; liver enzymes, bilirubin, and lipid profile were within normal limits (Table 1).

**Table 1:** Laboratory findings on admission CRP: C-reactive protein; AST: aspartate aminotransferase; ALT: alanine transaminase; ALP: alkaline phosphatase; GGT: gamma-glutamyl transferase

Parameter	Result	Reference Min	Reference Max	Unit
WBC	10,35	4	10	×10⁹/L
CRP	1,88	0	5	mg/L
Lipase	264,8	13	60	U/L
Amylase	201	28	100	U/L
AST	18.1	0	35	U/L
ALT	19.2	0	45	U/L
ALP	67	40	129	U/L
GGT	17.4	8	61	U/L
Total Bilirubin	0.41	0.2	1.2	mg/dL
Direct Bilirubin	0.11	0	0.3	mg/dL

**Figure 1:**
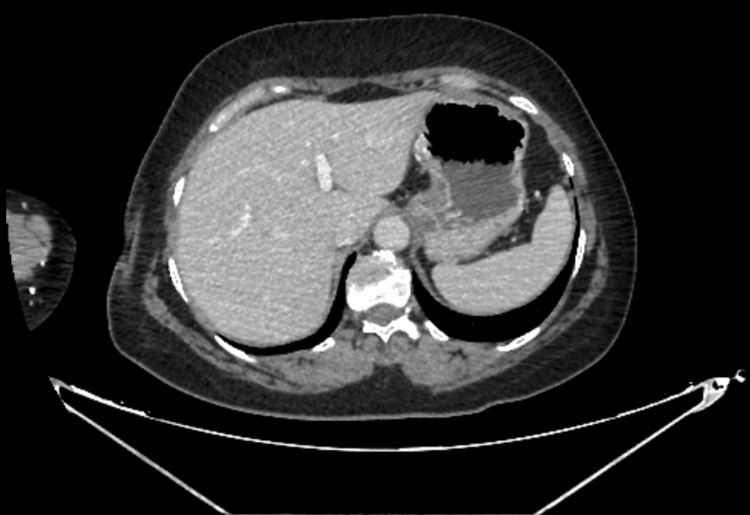
Wall thickening of the gastric antrum and pyloric segment

A contrast-enhanced CT scan of the abdomen was performed to clarify the symptoms. This revealed wall thickening in the antral and pyloric regions, as detected in Figure 1. This finding was consistent with the existing endoscopic findings, and the lower abdominal segments were found to be normal. There was no evidence of gallstones or other biliary tract disease that could explain the clinical presentation.

Although not stated in the report, the presence of hyperaemic and oedematous antral mucosa - together with chronic inflammation - shows focal oedema and bleeding, which are findings consistent with the wall thickening. The patient was discharged after four days with clinical improvement. Her lipase level had decreased to 37.1 U/L, and her pain - assessed using the numeric rating scale - had improved from 8/10 at admission to 1/10 at discharge.

At outpatient follow-up two weeks later, the patient was reported to have completely resolved her abdominal pain and vomiting, with no other gastrointestinal symptoms. Repeat laboratory tests revealed normal lipase (28 U/L) and amylase (41 U/L) levels, and liver enzymes remained within the normal range. A subsequent abdominal ultrasound revealed no peripancreatic fluid accumulation or structural abnormalities. The patient was advised to continue her proton pump inhibitor therapy and avoid factors that increase intra-abdominal pressure. During the subsequent six-week observation period, she experienced no further episodes of abdominal pain or pancreatitis.

## Discussion

Post-endoscopic pancreatitis is rarely described in the literature, particularly in patients without classic risk factors, such as gallstones, alcohol consumption, or elevated triglyceride levels. Published case reports vary considerably regarding the onset, severity (Table 2), and suspected causes of symptoms, suggesting that endoscopic pancreatitis can arise from various processes.

**Table 2:** Summary of reported cases of post-endoscopy acute pancreatitis

Parameter	Case 1	Case 2	Case 3	Case 4
Age/Sex	50-year-old male	69-year-old male	52-year-old male	46-year-old female
Procedure	Upper gastrointestinal endoscopy	Upper gastrointestinal endoscopy	Endoscopy + colonoscopy	Upper GI endoscopy + EMR of duodenal polyps
Onset of symptoms	Less than 2 days later, following endoscopy	4 hours after endoscopy	3 hours after procedures	24 hours post-procedure
Serum lipase (13–60 U/L)	5966 U/L	11,960 U/L	7060 U/L	2002 U/L
Serum amylase (30–110 U/L)	495 U/L	Not reported	Not reported	Not reported
Imaging findings	Pancreatic edema	Acute pancreatitis	Pancreatic edema	Acute pancreatitis with reactive gallbladder and duodenal changes
Proposed mechanism	Duodenal insufflation or mechanical irritation	Post-endoscopy pancreatitis	Polyp biopsy near ampulla of Vater	Ampullectomy/EMR near ampulla of Vater → mechanical irritation/injury
Source	[3]	[4]	[5]	[6]

In one report, a 50-year-old patient with Child-Pugh A liver cirrhosis developed abdominal pain and vomiting two days after endoscopy. Lipase levels were markedly elevated, and radiological examination revealed pancreatic edema. Possible mechanisms suggested included excessive duodenal insufflation or mechanical irritation during the procedure [3]. In another case, a 69-year-old man presented with back pain and vomiting four hours after endoscopy. His lipase level was also very high (11,960 U/L), suggesting a possible triggering effect of aggressive insufflation or transient papilledema [4]. A 52-year-old man developed pancreatitis with elevated lipase (7,060 U/L) and pancreatic edema on imaging three hours after upper gastrointestinal endoscopy and colonoscopy. In this case, the pancreatitis was associated with the biopsy of a polyp near the papilla of Vater [5]. A similar pattern was observed in a 46-year-old woman who developed pancreatitis 24 hours after excision of a single-cell intestinal polyp near the papilla of Vater [6].

Compared to these reports, our patients differ in several important ways. First, in our patient, the biopsy - unlike in cases where the biopsy or polypectomy was performed in the region of the papilla of Vater (a procedure that allows for direct mechanical irritation of the pancreatic ductal system) - was taken from the antrum and cardia, away from the pancreatic head. This reduces the risk of direct trauma to the papilla of Vater. Had the papilla of Vater been directly traumatized, this already rare entity - post-procedural acute pancreatitis - could have been considered a rare but relatively strong etiological factor from the outset [7]. Second, in our case, the symptoms appeared seven days after the endoscopy, a few hours to two days later than the typical onset described in most reports [8]. This delayed onset raises questions about indirect mechanisms, such as prolonged intraluminal pressure, temporary unicellular bowel immobilization, or peritoneal detachment resulting from the prolonged manipulation. Despite these differences, many factors associated with the procedure in this case are similar to those described in the literature. Both the prolonged duration of the procedure and technically demanding maneuvers - such as rotating the patient 45 degrees and repeated tissue sampling - can lead to increased single-cell dilatation or local mechanical stress. Continuous CO₂ infusion for 30 minutes can cause a transient increase in intracellular pressure, a mechanism described in several previous reports. In contrast to the previously described case, our patient underwent a comprehensive investigation for autoimmune, rheumatological, metabolic, infectious, and structural causes of pancreatitis, all of which were negative, further supporting the hypothesis of a process-related etiology.

Drug-induced pancreatitis has been described, particularly in association with amlodipine; however, a causal relationship was considered unlikely due to the short duration of treatment and the lack of documented cases. Vortioxetine was used for an extended period without any gastrointestinal or pancreatic adverse effects. Observations supporting drug-induced pancreatitis do not apply to our patient. Unlike the cases described in drug-induced acute pancreatitis (DIAP) cohorts, our patient had no significant comorbidities, no underlying malignancy or autoimmune disease, and no pre-existing organ dysfunction at the time of endoscopy. Therefore, the mechanism proposed for the increased severity of drug-induced pancreatitis cannot account for the clinical course observed in our case [9]. Unlike reports of patients with intrinsic risk factors such as liver cirrhosis, biliary tract disease, or peripapillary polypectomy, our patient had no pre-existing comorbidities, suggesting that only procedural factors may have played a role.

Although most published cases of post-endoscopic acute pancreatitis describe symptom onset within hours to 48 hours after the procedure, there are rare reports of late-onset pancreatitis occurring months after endoscopic transpapillary interventions [10]. To our knowledge, a seven-day delay in symptom onset after a purely diagnostic upper endoscopy without papillary manipulation has not been previously described.

Overall, there are similarities to previously described cases regarding longer procedure times, technical difficulties, and intra-abdominal pressure changes, while differences exist in the late onset of symptoms and the location of the procedure. These differences reflect the variability of post-endoscopic pancreatitis and underscore the importance of maintaining suspicion for pancreatitis even when the procedure does not involve the papilla.

## Conclusions

The most common causes of acute pancreatitis in this patient were ruled out, with the recent upper gastrointestinal endoscopy being the most likely cause. The lengthy duration of the procedure, the technical difficulty of polyp removal, the necessary 45-degree repositioning, and the prolonged CO₂ administration may have caused transient intraluminal pressure changes and local mechanical irritation. This case highlights the potential of endoscopies to trigger acute pancreatitis. This possibility should therefore be considered in cases of new abdominal pain or elevated pancreatic enzyme levels shortly after the procedure.
